# Supplementary material for: Exploration of Artificial Intelligence Use with ARIES in Multiple Myeloma Research
Source: J Clin Med. 2019 Jul 9;8(7):999. doi: 10.3390/jcm8070999 (PMC6678083; doi:10.3390/jcm8070999)
Supplement: Supplementary file 1 [file jcm-08-00999-s001.pdf]

# Supplementary Materials: Exploration of Artificial Intelligence Usage in Multiple Myeloma Research with ARIES

**Table S1**. Attributes Covered by Multiple Myeloma Ontology

| Parent Attribute | First Child Attribute | Second Child Attribute | Third Child Attribute |
|---|---|---|---|
| CRAB | Anemia | | |
| | Bone lesion | | |
| | Drop of Hb | | |
| | Hypercalcemia | | |
| | Renal impairment | | |
| | SLIM-CRAB | | |
| Cytogenetic | High risk cytogenetic | del(17/17p) | |
| | | gain(1q) | |
| | | t(14;16) | |
| | | t(14;20) | |
| | | t(4;14) | |
| | del(1p) | | |
| | del(13q) | | |
| | t(11;14) | | |
| | Hyperdiploidy | | |
| | Tetrasomy | | |
| | Trisomy | | |
| Laboratory | Free kappa light chain | | |
| | Free lambda light chain | | |
| | kappa light chain (SU) | | |
| | lambda light chain (SU) | | |
| | Beta 2 microglobulin | | |
| | CRP | | |
| | GFR | | |
| | Hemoglobulin | | |
| | Immunoglobulin A | | |
| | Immunoglobulin D | | |
| | Immunoglobulin G | | |
| | Immunoglobulin M | | |
| | Calcium | | |
| | Creatinin | | |
| | LDH | | |
| | Leucocytes | | |
| | Lymphocytes | | |
| | Neutrophiles | | |
| | Thrombocytes | | |
| | Total albumin level serum | | |
| | Total albuminuria level (SU) | | |
| | Total protein level serum | | |
| | Total proteinuria level (SU) | | |
| | Urea | | |
| | Type of myeloma | Asecretoric myeloma | |

| | | | |
|---|---|---|---|
| Myeloma specific | | Biclonal myeloma<br>Extramedullary myeloma<br>Light chain myeloma<br>Multiple myeloma<br>Monoclonal gammopathy of undetermined significance (MGUS)<br>Smoldering multiple myeloma<br>Solitary plasmocytoma | |
| | Bence Jones proteinuria<br>Bence Jones proteinemia<br>Primary diagnosis<br>Heavy chain type<br>Light chain type<br>Bone marrow infiltration<br>Paraprotein<br>Pathologiclal fracture<br>Serum light chain kappa/lambda ratio | | |
| | Gastrointestinal | Diarrhea<br>Hepatomegaly<br>Mucositis<br>Soor<br>Splenomegaly<br>Stomatitis | |
| | Hematological | Cellular change | Leucocytopenia<br>Depletion of lymphocytes<br>Neutropenia<br>Pancytopenia<br>Thrombocytopenia<br>Tricytopenia |
| | | Immune paresis<br>B symptoms<br>Graft versus host<br>Hyperviscosity<br>Paraneoplasia<br>Plasma cell leukemia | |
| | Infection, Reactivation | Cytomegalovirus<br>Febrile neutropenia<br>Herpes simplex virus<br>Influenza<br>Mycosis<br>Fungal pneumonie<br>Epstein–Barr virus<br>Hepatitis B virus<br>Respiratory syncytial virus<br>Superinfection<br>Varizalle zoster virus | |

| | | | |
|---|---|---|---|
| Adverse Events, Complications, Comorbidities | Kardiological | Cardiac arrhythmia | AV block |
| | | | Bradycardia |
| | | | Sick sinus syndrom |
| | | | Tachycardia |
| | | | Atrial fibrillation |
| | | | Atrial flutter |
| | | Cerebrovascular Occlusive Disease | |
| | | Endocarditis | |
| | | Congestive heart failure | |
| | | Valvular insufficiency | |
| | | Hypertensive crisis | |
| | | Myocarditis | |
| | | Pericarditis | |
| | | Syncope | |
| | | Venous thromboembolism | |
| | | Thrombosis | |
| | Nephrological | Acute kidney failure | |
| | | Amyloidosis | |
| | | CAST Nephropathia | |
| | | Chronic kidney failure | |
| | | Exacerbation of retention parameters | |
| | | Terminal kidney failure | |
| | Neurological | Compression of myelon | |
| | | Polyneuropathy | |
| | | Spinal canal stenosis | |
| | Admission on Intensive care unit | | |
| | Death on Intensive care unit | | |
| | Vertebral compression fracture | | |
| | Exanthema | | |
| | Fatigue | | |
| | Fever | | |
| | Cachexia | | |
| | Osteonecrosis of the jaw | | |
| | Adrenal insufficiency | | |
| | Pneumonia | | |
| | Sepsis | | |
| | Side effect | | |
| Patient | Age primary diagnosis | | |
| | ECOG | | |
| | Date of birth | | |
| | ICD 10 | | |
| | Karnofsky | | |
| Response criteria | CR | | |
| | MR | | |
| | NC | | |
| | nCR | | |
| | PD | | |
| | PR | | |

| | | |
|---|---|---|
| | sCR<br>Stable disease<br>VGPR | |
| Risk factors | Metabolic risk factors | Hypercholesterolemia<br>Hypertriglycerinemia<br>Dyslipoproteinemia<br>Hyperlipoproteinemia<br>Hypolipoproteinemia<br>Diabetes mellitus<br>Homocysteinemia |
| | Obesity<br>Smoker<br>Arterial hypertension<br>Coronary heart disease | |
| Staging | ISS<br>revised ISS<br>Salmon and Durie | |
| Therapy | Bisphosphonate therapy<br>Allogeneic stem cell transplantation<br>Autologous stem cell transplantation<br>Stem cell apheresis<br>Stem cell mobilisation<br>Start of therapy<br>Dose reduction<br>End of therapy<br>HD melphalan<br>Progression<br>Protocol<br>Radiation<br>Refractory<br>Relapse<br>Response | |
| Substances | Adriamycin<br>Bendamustin<br>Bortezomib<br>Carfilzomib<br>Carmustin<br>Cisplatin<br>Cyclophosphamid<br>Cytarabin<br>Daratumumab<br>Denosumab<br>Dexamethason<br>Doxorubicin<br>Elotuzumab<br>Etoposid<br>Ixazomib<br>Lenalidomid | |

| | |
|---|---|
| | Panobinostat<br>Pomalidomid<br>Prednisolon<br>Rituximab<br>Thalidomid<br>Vincristin |
